# Popliteal Artery Injury During Total Knee Replacement: A Single-Center Experience

**DOI:** 10.7759/cureus.71745

**Published:** 2024-10-17

**Authors:** Mohammad Alsaleem, Mohammed I Almomatten, Abdullah M Alkhars, Mohammed AlSaeed, Ali A Alsakkak

**Affiliations:** 1 Orthopedic Surgery, Al Moosa Specialist Hospital, Al-Ahsa, SAU; 2 Orthopedic Surgery, King Fahad Hospital Hofuf, Al-Ahsa, SAU; 3 Vascular Surgery, King Fahad Hospital Hofuf, Al-Ahsa, SAU; 4 College of Medicine, King Faisal University, Al-Ahsa, SAU

**Keywords:** arterial injury, orthopedic vascular complications, popliteal artery injury, total knee replacement, total knee replacement complications

## Abstract

The popliteal arteries are rarely injured during total knee replacement. Although uncommon, these complications warrant attention because the consequences can be disastrous, with high mortality rates, amputation risk, and additional comorbidities such as foot drop, infection, and functional impairment. However, it has detrimental consequences for the patient. Avoiding such injuries starts with preoperative planning, which helps prevent injuries. Furthermore, preoperative planning prompts early recognition and facilitates immediate interventions to reduce complications.

We presented the case of a 59-year-old female following up in an orthopedic outpatient clinic for advanced bilateral knee pain, which became more progressive in the past year. The patient had tried conservative treatment with minimal improvement and elected for total knee replacement. The operation was performed as standard. However, while trying to clear the posterior tissue, an accidental complete cut to the popliteal artery occurred. After that, the vascular surgeon performed direct repair of continuous anastomosis. Following that, distal pulses and capillary refill were felt by the surgeon to be intact. Finally, the operation was completed uneventfully. Later, the patient was discharged home in good condition and had no complications upon follow-up.

## Introduction

Around 40% of the global population over 55 suffers from persistent knee discomfort. Of the 40%, 50.8 million suffer from debilitating pain, with 2.6 million requiring total knee replacement (TKR) each year [1]. TKR has a shallow risk of vascular complications. A study of 39,196 TKR found that the most frequently injured large vessels were the popliteal artery (0.07%), the superficial femoral artery (0.02%), and the anterior tibial artery (0.005%) [2]. The overall prevalence varies between 0.003% and 0.23% [3-6]. A systematic review and meta-analysis study of vascular injuries in total knee arthroplasty stated that one out of five orthopedic surgeons would encounter a vascular injury associated with a TKR in their career [7].

The popliteal arteries are rarely injured during TKR. It is estimated to be 0.03-0.51% in case studies from single institutions or small multicenter studies [8-11]. Although uncommon, these complications warrant attention because the consequences can be disastrous, with mortality rates as high as 7%; amputation up to 42%; and additional comorbidities such as foot drop, infection, and functional impairment [2,9,12]. The prevention of vascular injuries can be accomplished with careful surgical technique and positioning of the knee during the procedure.

A thorough understanding of normal vascular branching patterns is important to prepare a surgeon to identify the potential sources of intraoperative popliteal artery injury, which may occur because of either blunt or sharp instruments. Rubash and his colleagues [13] studied popliteal artery damage risk zones during TKR. They identified the 12 o'clock position as the location of the popliteal vein, the 6 o'clock position as the most anterior point of the tibia, and the 1 o'clock position as the location of the popliteal artery. They found that between 11 and 3 o'clock, the insertion of retractors or the migration of the saw blade constituted a relative danger zone for injury. Although direct pressure and packing may be used to manage these injuries, most require immediate intraoperative evaluation by a vascular surgeon.

## Case presentation

**Figure 1:**
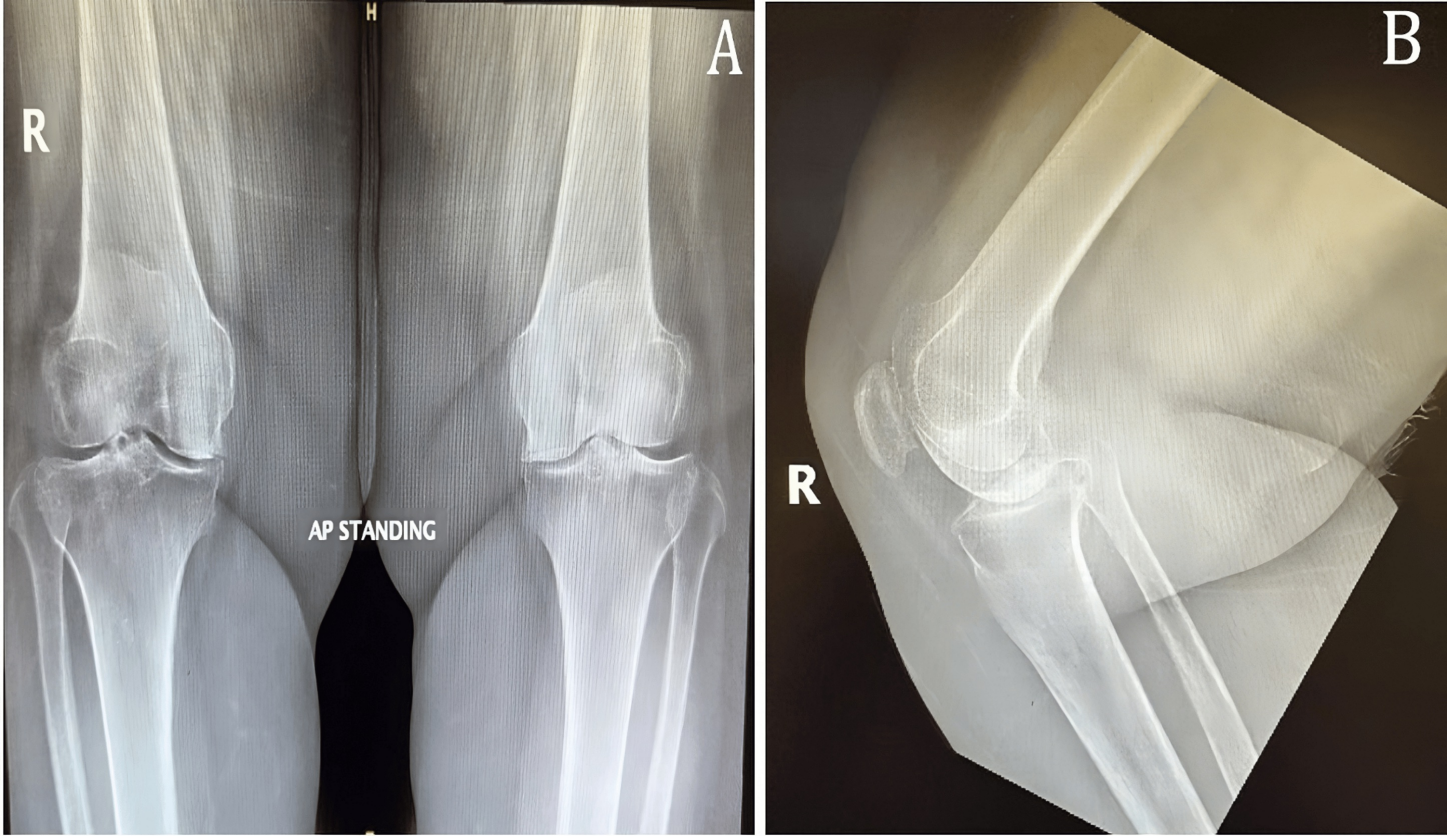
(A) Bilateral standing knee AP view X-ray preoperatively. (B) Right knee lateral view X-ray preoperatively AP: anteroposterior

A 59-year-old female patient presented to the orthopedics outpatient clinic complaining of chronic progressive, dull aching bilateral knee pain marked on the right side for more than five years. In the past year, she reported an increase in pain intensity with intermittent swelling, a significant limitation in her range of motion (ROM), and significant limitations in daily life activities. The patient has tried conservative management for three years but has shown minimal improvement. The patient is a known case of rheumatoid arthritis, sickle cell trait, and G6PD deficiency with no past surgical history. Upon examination, the patient's BMI was 33. She had an antalgic gait, bilateral knee genu varum deformity, and bilateral medial joint line tenderness. Moreover, she had 10 degrees of extension lag and knee flexion limited to <100 degrees. Meanwhile, her laboratory investigations were unremarkable. A bilateral knee X-ray showed bilaterally advanced knee osteoarthritis (Figure 1). 

**Figure 2:**
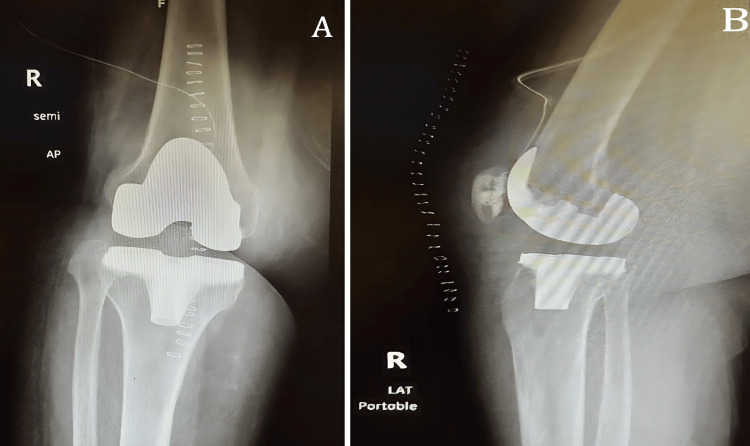
Right knee X-rays immediately postoperatively: (A) AP view and (B) lateral view AP: anteroposterior

Following that, the orthopedic surgeon counseled the patient for a right TKR and she agreed to that. A few weeks later, the patient underwent the procedure. The operation was performed using Zimmer Biomet's Persona (Warsaw, Indiana, United States), the personalized knee system. The operation was done under spinal anesthesia and a tourniquet in the supine position. A medial parapatellar approach was utilized. The operation was completed as standard, starting with the femur cuts and then the tibial cut with sizing of both and assessment of flexion and extension gaps. After that, femoral box, tibial preparation, and patellar preparation were done. However, while trying to clear the posterior tissue from the remnant synovitis, an accidental injury to the popliteal artery occurred. Once the injury was confirmed, a vascular surgeon was consulted, who did an exploration from the same approach and found a complete arterial cut with thrombosis to the distal end of the artery. A thrombectomy was done on both sides, and direct repair was performed by continuous anastomosis. Following that, distal pulses and capillary refill were felt to be intact. Finally, cemented implants were applied (femur size 5, narrow posterior stabilized, tibia D, patella 26 mm diameter, 7.5 mm thickness, polyethylene inserts 14 mm) with good flexion and extension gaps, stable varus, valgus stress, and patellofemoral tracking. Finally, the soft tissue and skin closed. The total blood loss from the procedure was 200 ml, and the patient received 1 unit of packed red blood cells (PRBCs) intraoperatively. The patient was examined postoperatively and found to be vitally stable with intact distal neurovascularity and capillary refill. Postoperative laboratory investigation showed no hemoglobin drop, and postoperative X-rays were obtained (Figure 2). One day postoperatively, the patient started her post-TKR physiotherapy protocol and was discharged home in good general condition. 

The patient was followed two weeks later, during which staples were removed. The patient was mobilizing well using a walker frame following her rehabilitation protocol and was found to have no joint swelling. Also, she had acceptable ROM (0-110) and intact distal neurovascularity. Standing X-rays taken showed good alignment with no implant malpositioning or sizing abnormalities.

Upon vascular follow-up, the ankle brachial pressure index was 1.0 which is normal, and Doppler ultrasonography for the distal arteries showed triphasic waves with intact pulsation and perfusion on the operated side. 

**Figure 3:**
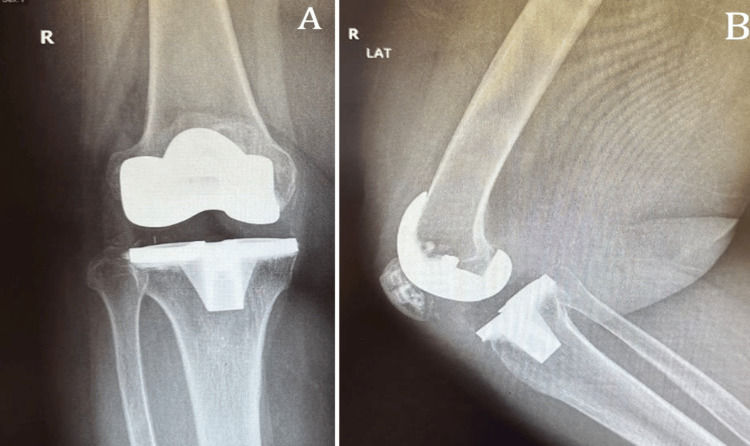
Right knee X-ray two months postoperatively: (A) AP view and (B) lateral view AP: anteroposterior

Two months follow-up, the patient had no active complaints of mobilizing without walking aids, a healthy surgical scar, and no tenderness. She had 120 degrees of flexion, full extension, stable varus and valgus stress, and patellofemoral tracking with intact distal neurovascular status. X-rays were obtained and showed well-fitting implants with no loosening (Figure 3). 

Six months postoperatively, the patient was doing fine and had good muscle strength with 130 degrees of flexion, full extension, stable varus and valgus stress, and patellofemoral tracking with intact distal neurovascular status.

**Figure 4:**
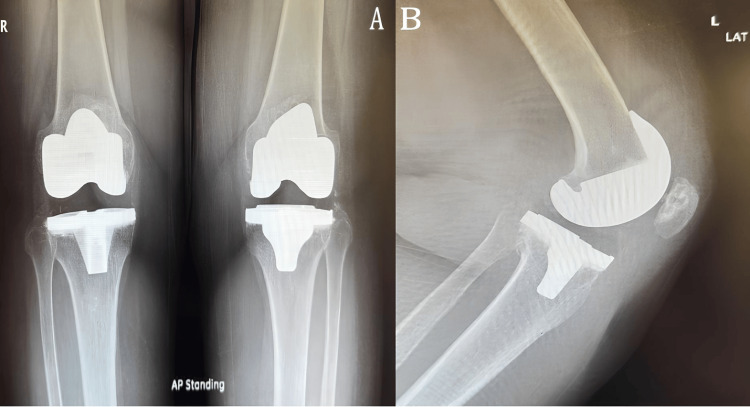
(A) Bilateral standing knee AP view X-ray two months postoperatively of the left side and 11 months operatively of the right side. (B) Left lateral view AP: anteroposterior

Eventually, the patient was happy and stated that her quality of life had improved significantly, especially in terms of pain, stiffness, and ROM. Fourteenthly, she came later, asking the surgeon to operate on her left side, which was done later and went smoothly with no complications, and she was satisfied with the outcome (Figure 4). 

This case has been reported in line with the Surgical Case Report (SCARE) 2020 guidelines [14].

## Discussion

Arterial injury after TKR is rare and has unsatisfactory consequences for both patient and surgeon. However, with the rise in TKR utilization in the aging population, the prevalence of these complications will likely increase as well. The most common location for TKR injury has been the popliteal artery, most likely due to traction injury or direct trauma. 

There are four moments of TKR during which the popliteal artery can be damaged: (a) a sharp injury to the popliteal artery may occur during a posterior cut of the femoral condyles, (b) a sharp injury to the popliteal artery may occur during tibial cuts, (c) a blunt injury may occur during the application of retractor for anterior dislocation of the tibia, and (d) an indirect traction injury may occur during the placement of the knee in hyperextension after the cuts and before the application of the hardware [15]. To recognize early vascular injury and facilitate early intervention, popliteal artery injuries can occur due to the vascular occlusion or embolization of calcified plaque from another site, usually from the area of tourniquet application. Sharp transection of the artery during bone cuts, arteriovenous fistula formation, and pseudoaneurysm formation are other ways the injury can also occur [6]. If it happens, intraoperative vascular consultation when recognizing arterial injury for emergent revascularization procedures to restore adequate limb perfusion distal to the site of vascular injury is required. A posterior approach to the popliteal fossa is recommended for these procedures, changing the patient to a prone position [16]. Our patient had a primary repair of a full-thickness anterior popliteal artery tear. Using the anterior approach facilitated by femoral and tibial osteotomies and knee in a flexed position, the vascular surgeon repaired the injured vessel. Moreover, the orthopedic surgeon proceeded with implant application and cementing without changing the position. 

Screening during outpatient visits before surgery for a history of vascular disease and physical examination helps identify at-risk patients and facilitates the prevention and early recognition of arterial injury. Vascular surgeon involvement in a multidisciplinary approach to patient care is important in such patients who may require preoperative arterial studies or revascularization procedures before TKR [16]. 

Moreover, postoperative monitoring by physical examination, pulse monitoring, and perfusion of the operated limp is needed to exclude vascular injury. Also, patient-specific complaints such as unusual and persistent posterior knee pain, swelling, and paresthesia may raise red flags for further investigations and interventions. Finally, it is important to assess each patient to implement a safe approach to each surgery and prompt early intervention to avoid detrimental surgical complications.

## Conclusions

Arterial injury in total knee arthroplasty is rare. However, it has detrimental consequences for the patient. Avoiding such injuries starts with preoperative planning, which helps prevent injuries. Furthermore, preoperative planning prompts early recognition and facilitates immediate interventions to reduce complications.

## References

[1] (2021). Demand for knee replacement grows 5 percent worldwide. https://orthospinenews.com/2019/06/04/demand-for-knee-replacement-grows-5-percent-worldwide/.

[2] Troutman DA, Dougherty MJ, Spivack AI, Calligaro KD (2013). Updated strategies to treat acute arterial complications associated with total knee and hip arthroplasty. J Vasc Surg.

[3] Ko LJ, DeHart ML, Yoo JU, Huff TW (2014). Popliteal artery injury associated with total knee arthroplasty: trends, costs and risk factors. J Arthroplasty.

[4] Rand JA (1987). Vascular complications of total knee arthroplasty. J Arthroplasty.

[5] Bernhoff K, Rudström H, Gedeborg R, Björck M (2013). Popliteal artery injury during knee replacement: a population-based nationwide study. Bone Joint J.

[6] Papadopoulos DV, Koulouvaris P, Lykissas MG, Giannoulis D, Georgios A, Mavrodontidis A (2015). Popliteal artery damage during total knee arthroplasty. Arthroplast Today.

[7] Sundaram K, Udo-Inyang I, Mont MA, Molloy R, Higuera-Rueda C, Piuzzi NS (2020). Vascular injuries in total knee arthroplasty: a systematic review and meta-analysis. JBJS Rev.

[8] Inomata K, Sekiya I, Otabe K (2017). Acute arterial occlusion after total knee arthroplasty: a case report. Clin Case Rep.

[9] Holmberg A, Milbrink J, Bergqvist D (1996). Arterial complications after knee arthroplasty: 4 cases and a review of the literature. Acta Orthop Scand.

[10] Abularrage CJ, Weiswasser JM, Dezee KJ, Slidell MB, Henderson WG, Sidawy AN (2008). Predictors of lower extremity arterial injury after total knee or total hip arthroplasty. J Vasc Surg.

[11] Calligaro KD, DeLaurentis DA, Booth RE, Rothman RH, Savarese RP, Dougherty MJ (1994). Acute arterial thrombosis associated with total knee arthroplasty. J Vasc Surg.

[12] Avisar E, Elvey MH, Bar-Ziv Y, Tamir E, Agar G (2015). Severe vascular complications and intervention following elective total hip and knee replacement: a 16-year retrospective analysis. J Orthop.

[13] Rubash HE, Berger RA, Britton CA, Nettrour WS, Seel MJ (1993). Avoiding neurologic and vascular injuries with screw fixation of the tibial component in total knee arthroplasty. Clin Orthop Relat Res.

[14] Agha RA, Franchi T, Sohrabi C, Mathew G, Kerwan A (2020). The SCARE 2020 guideline: updating consensus Surgical CAse REport (SCARE) guidelines. Int J Surg.

[15] Ninomiya JT, Dean JC, Goldberg VM (1999). Injury to the popliteal artery and its anatomic location in total knee arthroplasty. J Arthroplasty.

[16] Ogbu VO, Ugochukwu U, Oluwo A, Ekwele K, Ogedegbe F (2023). Popliteal artery injury following primary total knee replacement: a case report. East Afr Orthop J.

